# Personality Pathology Predicts Outcomes in a Treatment-Seeking Sample with Bipolar I Disorder

**DOI:** 10.1155/2014/816524

**Published:** 2014-01-02

**Authors:** Susan J. Wenze, Brandon A. Gaudiano, Lauren M. Weinstock, Ivan W. Miller

**Affiliations:** Alpert Medical School of Brown University and Butler Hospital, Psychosocial Research, 345 Blackstone Boulevard, Providence, RI, 02906, USA

## Abstract

We conducted a secondary analysis of data from a clinical trial to explore the relationship between degree of personality disorder (PD) pathology (i.e., number of subthreshold and threshold PD symptoms) and mood and functioning outcomes in Bipolar I Disorder (BD-I). Ninety-two participants completed baseline mood and functioning assessments and then underwent 4 months of treatment for an index manic, mixed, or depressed phase acute episode. Additional assessments occurred over a 28-month follow-up period. PD pathology did not predict psychosocial functioning or manic symptoms at 4 or 28 months. However, it did predict depressive symptoms at both timepoints, as well as percent time symptomatic. Clusters A and C pathology were most strongly associated with depression. Our findings fit with the literature highlighting the negative repercussions of PD pathology on a range of outcomes in mood disorders. This study builds upon previous research, which has largely focused on major depression and which has primarily taken a categorical approach to examining PD pathology in BD.

## 1. Introduction

Research suggests that comorbid personality disorder (PD) pathology has negative repercussions across a range of psychological disorders and for a variety of outcomes [1]. Within this body of literature, affective disorders have received a great deal of attention, with perhaps the largest number of studies focusing on major depressive disorder (MDD). Although some findings have been inconsistent [2], the majority of research has found adverse effects of PD pathology on the course of MDD [3–6] and on functional and symptomatic outcomes [7–11].

Relatively fewer studies have explored the impact of PD pathology on outcomes in bipolar disorder (BD), despite published comorbidity rates that range from 12% to 89% [12]. Overall, the literature suggests similar patterns as are evident in MDD. Compared to those without comorbid PD diagnoses, patients with BD and comorbid PDs have higher rates of hospitalization [13], suicide attempts and ideation [14, 15], psychosocial service utilization [16], and alcohol and substance abuse [17, 18]. They also have shown worse functional and symptomatic outcomes [19, 20] and poorer medication compliance and response [13, 21] in some studies.

Given overlapping clinical features, a number of studies have focused specifically on the effects of comorbid borderline personality disorder (BPD) in individuals with BD. Compared to those without such comorbidity, patients with BD and BPD have higher rates of psychotic symptoms during mood episodes [22], longer mood episodes [23], and worse medication adherence and response [22, 24]. Patients with comorbid cluster B (i.e., antisocial, histrionic, borderline, and narcissistic) PDs in general also have higher rates of suicide attempts than those without such comorbidity [25], and there is some evidence that cluster B diagnoses are more strongly associated with suicide attempts than cluster A (i.e., paranoid, schizoid, and schizotypal) or C (i.e., avoidant, dependent, and obsessive-compulsive) diagnoses [14]. However, clusters A and C comorbidity have also been found to confer risk for negative outcomes. Specifically, cluster A (but not B or C) symptoms were associated with long-term clinical status (euthymic versus symptomatic) in one study [26], whereas cluster C (but not A or B) symptoms were associated with residual depression severity in another study [20].

In sum, then, research suggests that PDs are associated with a range of negative outcomes amongst individuals with BD. However, we know little about how *degree* of personality pathology predicts outcomes in BD populations; the vast majority of studies have used categorical measures of PD pathology (i.e., assignment of a PD diagnosis or not) rather than dimensional measures (e.g., number of PD symptoms), despite the fact that the PD literature strongly favors a dimensional approach [27]. Results of those studies that have taken a dimensional approach suggest that degree of personality pathology negatively impacts long-term symptomatic and functional outcomes [20, 26, 28, 29]. This is consistent with findings from the unipolar depression literature [7].

In addition, it is unclear whether PD pathology, even when defined dimensionally, is associated with outcomes above and beyond other potentially important predictors. For example, few studies have controlled for baseline or recent mood symptom severity or functioning. In the case of data drawn from clinical trials, authors have not always specified whether they accounted for possible treatment condition effects. Importantly, given considerable comorbidity between PDs (particularly clusters C PDs) and anxiety disorders [30], as well as suggestions that avoidant personality disorder and social phobia represent overlapping constructs [31, 32], it would seem important to control for the presence of comorbid anxiety disorders in relevant analyses. However, we are not aware of any reports that have done so. Other methodological weaknesses in prior work have included the use of self-report measures to assess personality pathology, mood, or functioning, and combining participants with Bipolar I Disorder (BD-I) and Bipolar II Disorder in a single sample.

In the present investigation we conducted a secondary analysis of data from a clinical trial to explore the relationship between total number of PD symptoms and short and long-term mood and functioning outcomes in a treatment-seeking sample with BD-I. We controlled for clinically relevant variables in all analyses and conducted exploratory analyses on clusters A, B, and C pathology when appropriate. Since the literature suggests that personality pathology is more strongly associated with depressive symptomatology than with manic symptomatology [14, 15, 20, 25], we hypothesized that overall degree of personality pathology would predict depressive (but not manic) symptoms after acute treatment of a BD-I mood episode (i.e., at 4-month followup) and at long-term followup (i.e., at 28 months). We also expected that personality pathology would predict functional impairment at both timepoints. Finally, we expected that personality pathology would predict symptomatic status (i.e., percent time symptomatic) over the course of the 28-month follow-up period. When overall degree of personality predicted an outcome, we conducted exploratory analyses investigating the impact of clusters A, B, and C symptomatology on the outcome. However, given lack of consistency in previous research about which clusters are most problematic [14, 20, 26], we did not have any a priori hypotheses for these analyses.

## 2. Materials and Methods

### 2.1. Participants

Ninety-two participants were recruited for the parent study from inpatient, partial hospital, and outpatient settings at a university-affiliated psychiatric hospital. At the time of study enrollment (1993–1997), participants were required to meet Structured Clinical Instrument for DSM-III-R-Patient Version (SCID-I [33]) criteria for a current BD-I manic, depressed, or mixed mood episode. DSM-III-R and DSM-5 criteria for BD are virtually identical. Additional inclusion criteria are described in detail elsewhere [34]. Briefly, participants were required to (1) be between 18 and 75 years of age, (2) have adequate reading skills for completion of self-report measures, and (3) live with or be in regular contact with a relative or significant other. Participants were excluded if (1) they met DSM-III-R criteria for alcohol or drug dependence during the past year or a mood disorder secondary to a medical condition, (2) they had a medical condition that would preclude use of mood-stabilizing medication, or (3) they were pregnant or did not use adequate contraception (in females of child-bearing age).

Participants ranged in age from 18 to 73 years old (*M* = 39.57, SD = 11.30). Fifty-two participants (56.5%) were female and 40 (43.5%) were male. The majority of participants (*N* = 86; 93.5%) were Caucasian. Three participants (3.3%) were African American and one (1.1%) was Hispanic. Sixty-two participants (67.4%) were married or cohabiting and 30 (32.6%) were divorced, separated, or never married. Years of education ranged from 7 to 20 (*M* = 13.33, SD = 2.47). Sixty-nine participants (75%) were in a manic episode at the time of study entry, 18 (19.6%) were in a depressive episode, and 5 (5.4%) were in a mixed episode. Age of BD onset ranged from 6 to 61 years old (*M* = 23.65, SD = 10.43).

### 2.2. Procedure

The present study was part of a larger investigation; procedures are described in detail elsewhere [34–36]. Briefly, participants were approached during an acute mood episode to determine willingness to participate in an IRB-approved clinical trial. Following informed consent procedures, participants completed baseline assessments and were then randomized to receive acute treatment for BD lasting 4 months. Treatment conditions included (unstandardized) pharmacotherapy alone (i.e., treatment as usual), pharmacotherapy plus multifamily psychoeducational group therapy, or pharmacotherapy plus family therapy. Participants completed follow-up assessments on a monthly basis for the next 28 months. Assessments were attempted even if participants relapsed or discontinued participation in study treatment.

Of relevance for the present study, participants completed the diagnostic assessment (SCID-I [33]) at baseline, mood assessments at baseline, and at the 28 monthly follow-ups, functioning assessments at baseline and at the 2, 4, 10, 16, 22, and 28-month follow-up timepoints, and assessment of PD pathology 6 weeks after baseline. PD pathology was evaluated at this time point based on expert recommendations about minimizing the impact of acute mood symptoms on PD assessment [37] and based on the need to identify a time point by which acute mood symptoms had begun to decrease but which was still relatively close to baseline assessments [38]. Of note, recent evidence suggests that personality pathology can be validly assessed even in the presence of significant affective symptoms [8].

Data on PD pathology were available for 65 participants (70.65% of the total sample). Of these, data from the end of the acute treatment phase (i.e., 4-month followup) were available for 60 participants, and data from the final (i.e., 28-month) followup were available for 41 participants. Participants completed mood assessments on an average of 17.02 (SD = 11.31; 60.79%) of the 28 possible monthly assessments.

### 2.3. Measures

Bachelor's or master's-level research assistants administered all interview-based assessments. Prior to conducting assessments, research assistants received didactic training on all instruments and completed practice interviews until they reached at least 90% agreement with the PhD-level project coordinator. Reliability checks occurred regularly. Alpha remained above 0.85 at all times.

#### 2.3.1. Personality Pathology

The Structured Clinical Interview for DSM-III-R Personality Disorders (SCID-II [33]) was used to assess PD pathology. We operationalized degree of personality pathology as the total number of PD symptoms that met subthreshold or threshold levels for the 10 PDs that are included in the DSM-5 (i.e., paranoid, schizoid, schizotypal, antisocial, histrionic, borderline, narcissistic, avoidant, dependent, and obsessive-compulsive PDs). Differences between DSM-III-R and DSM-5 diagnostic criteria for these 10 PDs are minor. This dimensional approach is consistent with the literature suggesting that PD dimensions are more reliable, more stable, and more strongly associated with psychosocial morbidity than categorical diagnoses [27] and that a dimensional approach to *subthreshold* PD symptoms may be particularly important [39–41]. Of note, a 3-point dimensional approach toward diagnosing PDs, such as was captured in DSM-III-R and DSM-IV-based structured clinical interviews (i.e., absent, subthreshold, or threshold symptoms [42]), and as will presumably also be captured in structured clinical interviews based on DSM-5, has been shown to be equally valid as more finely-grained approaches [27].

#### 2.3.2. Mood Symptoms

Severity of depression was assessed via the Modified Hamilton Rating Scale for Depression (MHRSD [43]), a 17-item, interview-based measure. Severity of mania was assessed via the Bech-Rafaelsen Mania Scale (BRMS [44]), an 11-item, interview-based measure. Total scores can range from 0 to 50 for the MHRSD and 0 to 44 for the BRMS, with higher scores representing more severe symptoms on both measures. The MHRSD and the BRMS are widely used measures with established psychometric properties [43, 45].

#### 2.3.3. Symptomatic Status

We used the MHRSD and the BRMS to calculate percent time in-episode (i.e., fully symptomatic). At each follow-up assessment, participants were classified as symptomatic if they scored ≥ 15 on the BRMS and/or the MHRSD. This is consistent with published guidelines for these measures [46–48]. We then computed percent time symptomatic over the course of the 28-month follow-up period. Of note, we only calculated percent time symptomatic for participants who had at least 9 months of follow-up data (*N* = 63).

#### 2.3.4. Psychosocial Functioning

Psychosocial functioning was assessed via the UCLA Social Attainment Scale (SAS [49]). This 7-item interview-based measure yields a total score and three subscale scores, reflecting functioning related to peer relationships, romantic relationships, and involvement in activities. For the present study we used only the total score, which can range from 7 to 35, with higher scores representing better functioning. The SAS has been used previously in BD samples [20, 50–52].

## 3. Results and Discussion

### 3.1. Overview and Descriptive Analyses

All analyses were conducted using SPSS 20.0. We conducted hierarchical multiple regression analyses to determine the relationship between overall personality pathology (i.e., the total number of PD symptoms that met subthreshold or threshold levels) and outcome variables (manic and depressive symptoms, psychosocial functioning, and symptomatic status). If overall personality pathology was related to an outcome variable, we conducted exploratory analyses in which clusters A, B, and C symptoms were entered as simultaneous predictors of that outcome variable.

We controlled for treatment condition (using Helmert contrast codes to account for the 3 conditions) and presence of a comorbid anxiety disorder (per DSM-5; e.g., obsessive-compulsive disorder, posttraumatic stress disorder, and acute stress disorder are no longer counted as anxiety disorders) in all hierarchical multiple regression analyses. DSM-5 anxiety disorder diagnostic criteria are largely the same as DSM-III-R criteria. We also controlled for baseline mood symptoms and functioning in all relevant analyses. Age at BD onset was significantly related to psychosocial functioning at 4-month followup (*F*(1,64) = 5.11, *P* = 0.03) and percent time symptomatic (*F*(1,61) = 11.01, *P* < 0.01). Marital status (i.e., married or cohabiting versus divorced, separated, or never married) was significantly related to psychosocial functioning at 4 (*F*(1,64) = 28.95, *P* < 0.001) and 28-month (*F*(1,40) = 9.95, *P* < 0.01) followups. Therefore, in addition to treatment condition, comorbid anxiety, and baseline functioning or symptoms, we also controlled for age at BD onset and/or marital status in relevant analyses. No other baseline or demographic variables were related to our outcome variables.

Means, standard deviations, and intercorrelations between study variables are presented in Table 1. As expected, total number of PD symptoms was positively associated with symptoms in each of the 3 PD clusters, and with many of our symptom measures. Results of our main analyses are presented in Table 2. Participants for whom 4-month outcome data were available did not differ from those for whom such data were unavailable on baseline measures or overall degree of personality pathology (all *P* > 0.21). Similarly, participants for whom 28-month outcome data were available did not differ from those for whom such data were unavailable on these measures (all *P* > 0.06). Finally, participants who had at least 9 months of follow-up data did not differ from those who had 8 months or less of follow-up data on these measures (all *P* > 0.08).

Although we took a dimensional approach to personality pathology, we recognize that it would be informative to provide some descriptive information about threshold-level PD symptomatology in our sample. Number of PD diagnoses amongst study participants ranged from 0 to 2 (*M* = 0.08, SD = 0.32). The majority of participants who completed the SCID-II (*N* = 61; 93.85%) had no PD diagnoses. Number of threshold-level PD symptoms ranged from 0 to 21 (*M* = 5.03, SD = 5.00). Number of subthreshold PD symptoms ranged from 0 to 17 (*M* = 4.21, SD = 4.26).

### 3.2. Mood Symptoms

Results from analyses of manic and depressive mood symptoms are presented in Table 2. Controlling for treatment condition, presence of a comorbid anxiety disorder, and baseline manic symptoms, PD pathology did not predict manic symptoms at 4- or 28-month followup. Controlling for treatment condition, presence of a comorbid anxiety disorder, and baseline depressive symptoms, PD pathology predicted depressive symptoms at 4-month followup. When entered as simultaneous predictors, clusters A and B were not associated with depressive symptoms, but cluster C was. Controlling for treatment condition, presence of a comorbid anxiety disorder, and baseline depressive symptoms, PD pathology also predicted depressive symptoms at 28-month followup. When entered as simultaneous predictors, cluster A was associated with depressive symptoms, but clusters B and C were not.

For exploratory purposes, we tested the predictive role of personality pathology within each of the 3 PD diagnostic categories that comprise cluster C on depressive symptoms at 4-month followup and each of the 3 PD diagnostic categories that comprise cluster A on depressive symptoms at 28-month followup. When entered as simultaneous predictors and controlling for the previously noted variables, avoidant personality pathology was associated with depressive symptoms at 4 months (*β* = 0.43, *P* < 0.01, *sr* ^2^ = 0.12), but obsessive-compulsive and dependent personality pathology were not (*P*'s > 0.10). Schizotypal personality pathology was associated with depressive symptoms at 28 months (*β* = 0.54, *P* < 0.01, *sr* ^2^ = 0.24) but paranoid and schizoid personality pathology was not (*P*'s > 0.10).

### 3.3. Psychosocial Functioning

Controlling for treatment condition, presence of a comorbid anxiety disorder, baseline functioning, age at BD onset, and marital status, PD pathology did not predict psychosocial functioning at 4- or 28-month followup (see Table 2).

### 3.4. Symptomatic Status

Controlling for treatment condition, presence of a comorbid anxiety disorder, baseline depressive and manic symptoms, and age at BD onset, PD pathology predicted percent time symptomatic over the course of the 28-month follow-up period. When entered as simultaneous predictors, clusters A, B, and C were not independently associated with percent time symptomatic (see Table 2).

## 4. Discussion

In the current study we report results of secondary analyses on data from a clinical trial of treatment for an acute BD-I mood episode. We conducted a longitudinal assessment of the associations between PD pathology and symptom and functioning outcomes. Degree of PD pathology (i.e., total number of PD symptoms that met subthreshold or threshold levels) predicted depressive symptoms at the end of the acute treatment phase (i.e., 4-month followup) as well as at the final (28-month) followup, over and above shared variance with baseline depressive symptoms and other clinically relevant variables; a higher number of PD symptoms were associated with higher depression scores at both timepoints, suggesting a “dose-response” relationship between these variables. Importantly, although follow-up depression scores were relatively low, research clearly indicates that residual depressive symptoms are both common [53] and disabling [54, 55] amongst individuals with BD. Cluster C (i.e., avoidant, dependent, and obsessive-compulsive) pathology emerged as particularly problematic in terms of 4-month depressive symptoms, whereas cluster A (i.e., paranoid, schizoid, and schizotypal) pathology was most problematic in terms of 28-month depressive symptoms. PD pathology also predicted percent time symptomatic over the course of the 28-month follow-up period; a higher number of PD symptoms was associated with more time spent in-episode. Personality pathology was not associated with psychosocial functioning or manic symptoms at any timepoints.

Our findings fit with the overall body of literature highlighting the negative repercussions of comorbid personality pathology on a broad range of outcomes amongst individuals with mood disorders [9, 12]. This study builds upon previous research, which has largely focused on MDD and long-term course/outcomes [4, 8]. In addition, most previous research in this area has used a naturalistic or retrospective design; few have taken a prospective approach. Finally, and most importantly, the present study advances this body of literature by conceptualizing PD pathology from a dimensional perspective; previous research has primarily taken a categorical approach to examining personality pathology in BD [17, 19].

The fact that PD symptoms predicted follow-up depression scores but not mania scores might be due in part to insufficient variability in mania symptoms at follow-up timepoints; mania scores in our sample were relatively low at follow-up assessments. Nevertheless, these findings are consistent with previous research, which has underscored the association between personality pathology and bipolar *depressive* symptomatology [14, 15, 20, 25]. It could be that PD symptoms interfere with recovery from depression [56]; PD pathology might obstruct the therapeutic relationship, impede working alliance with treatment providers, or restrict the availability of (or the patient's ability to take advantage of) social support. Alternatively, depressive and PD symptoms may arise from shared processes [57], whereas the etiology of mania may be unrelated. In either case, personality pathology does not appear to be as strongly linked with manic symptoms.

Our failure to replicate the association between PD pathology and functional impairment in BD [19] may be due in part to the specific measure of psychosocial functioning we employed; the SAS assesses a somewhat limited number of domains of functioning and largely addresses interpersonal relationships. Most previous studies that have demonstrated a relationship between PDs and functional impairment in BD have focused on a broader range of domains or on occupational impairment, specifically [17, 19, 29, 58]. In addition, the majority of our participants (80.4%) were in a manic or mixed episode at the time of study entry. Research has suggested that depression, but not mania, prospectively predicts functional impairment in BD [59–61].

The current study highlights the importance of assessing personality pathology dimensionally, rather than categorically. Although this approach is widely accepted amongst PD researchers as being preferable [27], only a handful of studies focusing specifically on PD pathology in BD have recognized and utilized this method [26]. In the present sample, the majority of participants did not have a PD *diagnosis*. However, PD *symptoms* were prevalent and, as noted, prospectively predicted symptoms of depression and percent time in-episode. These findings suggest that therapeutic attention to subsyndromal PD symptomatology may be necessary in order to improve clinical outcomes in the acute treatment of a BD-I mood episode. Our results also underscore the importance of utilizing a variety of treatment strategies in working with BD. For example, optimal treatment following a mood episode might entail not only pharmacotherapy to ameliorate acute mood symptoms but also psychotherapy to address longer-term problems, such as interpersonal conflict, maladaptive thought patterns, or negative coping strategies. These types of difficulties, which are characteristic of PD pathology, are unlikely to be helped by pharmacotherapy alone.

In this study, clusters A and C pathology emerged as particularly problematic in terms of follow-up depression scores. The relationship between cluster C symptoms and longitudinal outcome is particularly striking given that we controlled for comorbid anxiety disorders. It is possible that symptoms such as suspicion over others' motives or avoidance of interpersonal engagement, as might be found in individuals with subsyndromal features of cluster A or C, respectively, could interfere with treatment received in the context of a clinical trial. Given that few previous studies have explored which PD clusters or diagnoses are most predictive of poor outcomes in BD and those that have examined this have yielded inconsistent results [14, 20, 26], future research should continue to explore this issue.

Future studies should also address limitations of the present research, which include a relatively modest and predominantly Caucasian sample and participants who were primarily in manic or mixed episodes at the time of study entry. It will be important to determine whether similar results are obtained in samples that are more demographically and symptomatically diverse. In particular, it will be worthwhile to determine whether results are comparable in samples that are predominantly (or exclusively) in the depressive phase of illness. Results should also be replicated in a sample meeting DSM-5 criteria for BD-I (and in which PD symptoms and presence of anxiety disorders are assessed according to DSM-5 criteria) although, as noted, DSM-III-R and DSM-5 criteria for these disorders are very similar so we would not anticipate significant changes in our findings. As previously discussed, our measure of psychosocial functioning assessed a limited number of domains and focused largely on interpersonal relationships; future research should determine whether PD pathology is associated with psychosocial functioning, as assessed by different measures.

Finally, our sample included very few participants who had a PD diagnosis. Although this is not problematic when PD pathology is conceptualized from a dimensional perspective, as was the case in the present study, the comorbidity rate observed in our sample is not consistent with what has been reported in previous research [12]. One possible explanation for this difference is that participants in the current study were required to be living with or in regular contact with a relative or significant other; individuals with this level and type of interpersonal connection might be less likely to meet criteria for a PD diagnosis. Further, data on PD pathology was only available for 71% of our sample; data from the remaining 29% of participants might have resulted in greater rates of PD comorbidity that is more comparable to previous reports. In any case, total number of PD symptoms was clearly associated with prospective depressive symptoms and total amount of time spent in-episode in the present investigation, even after controlling for baseline symptoms, treatment condition, comorbid anxiety disorders, and age at BD onset. An even stronger relationship might be evident if more participants with PD diagnoses had been included. Additional studies using samples with higher PD comorbidity rates will help to address this question.

## 5. Conclusions

The results of the present study highlight the importance of conceptualizing PD pathology from a dimensional perspective, at least when considering its impact on outcomes in BD. Data revealed that degree of PD pathology (i.e., number of subthreshold and threshold-level PD symptoms) prospectively predicts depressive symptoms and percent time symptomatic amongst individuals with BD-I. Clusters A and C pathology were most strongly associated with depression severity. It is striking that these results held even when controlling for other potentially important predictors, such as treatment condition, presence of comorbid anxiety disorders, and baseline symptoms and functioning. Our findings fit with the literature highlighting the negative repercussions of PD pathology on a range of outcomes in mood disorders. This study builds upon previous research, which has largely focused on MDD and which has primarily taken a categorical approach to examining PD pathology in BD. Future research will aid in determining whether these findings are similar in more demographically, symptomatically, and diagnostically diverse populations.

## Figures and Tables

**Table 1:** Means, standard deviations, and intercorrelations between study variables.

Variable	*M *	SD	Correlation matrix^a^												
			2	3	4	5	6	7	8	9	10	11	12	13	14
(1) Baseline MHRSD	9.09	9.28	−0.69**	−0.10	0.23	0.20	−0.01	0.10	0.07	0.09	0.46**	0.38**	0.20	0.44**	0.36**
(2) Baseline BRMS	21.41	10.52	—	0.20	−0.10	−0.09	0.02	0.04	−0.08	−0.18	−0.27*	−0.26*	−0.13	−0.22	−0.30*
(3) Baseline SAS	23.72	6.09		—	−0.30*	−0.06	0.79**	0.09	0.28	0.68**	−0.17	0.03	−0.01	0.06	−0.05
(4) 4-month MHRSD	8.62	6.89			—	0.15	−0.34**	0.54**	0.05	−0.33*	0.58**	0.41**	0.23	0.35**	0.43**
(5) 4-month BRMS	3.41	5.12				—	−0.00	0.05	0.18	0.09	0.48**	0.11	0.02	0.08	0.12
(6) 4-month SAS	22.82	5.62					—	−0.13	0.10	0.63**	−0.19	0.05	−0.16	0.11	−0.01
(7) 28-month MHRSD	5.83	5.21						—	0.21	−0.10	0.49**	0.33*	0.47*	0.23	0.15
(8) 28-month BRMS	1.81	3.36							—	0.28	−0.06	−0.00	0.21	−0.11	0.05
(9) 28-month SAS	26.48	7.15								—	0.06	0.21	0.08	0.22	0.10
(10) Percent time symptomatic	0.25	0.30									—	0.47*	0.40**	0.51**	0.45**
(11) Total number of PD symptoms	9.16	7.92										—	0.58**	0.82**	0.84**
(12) Total number cluster A symptoms	1.30	1.83											—	0.25*	0.32**
(13) Total number cluster B symptoms	2.98	3.74												—	0.53**
(14) Total number cluster C symptoms	3.74	3.38													—

*P< 0.05. **P< 0.01.

^a^Numbers correspond to the numbered variables in the left-most column of the table.

**Table 2:** Hierarchical multiple regression analyses predicting psychosocial functioning, manic and depressive symptoms, and percent time symptomatic from personality pathology.

	Psychosocial functioning			
	4-month followup		28-month followup	
Predictor: PD pathology	*β*	sr^2^	*β*	sr^2^
Overall	−0.03	0.00	0.10	0.01

	Manic symptoms			
	4-month followup		28-month followup	

Predictor: PD pathology	*β*	sr^2^	*β*	sr^2^
Overall	0.12	0.01	0.02	0.00

	Depressive symptoms			
	4-month followup		28-month followup	

Predictor: PD pathology	*β*	sr^2^	*β*	sr^2^
Overall	0.47**	0.16	0.41*	0.14
Cluster A^a^	0.12	0.01	0.47**	0.19
Cluster B^a^	0.17	0.02	0.26	0.03
Cluster C^a^	0.36*	0.09	−0.09	0.00

	Percent time symptomatic			

Predictor: PD pathology	*β*		sr^2^	
Overall	0.27*		0.06	
Cluster A^b^	0.15		0.02	
Cluster B^b^	0.16		0.01	
Cluster C^b^	0.28^†^		0.04	

^†^ *P*< 0.10. *P< 0.05. **P< 0.01.

^a^Clusters were entered as simultaneous predictors.

^b^Clusters were entered as simultaneous predictors.
